# Low human and murine Mcl-1 expression leads to a pro-apoptotic plaque phenotype enriched in giant-cells

**DOI:** 10.1038/s41598-019-51020-3

**Published:** 2019-10-10

**Authors:** Margaux A. C. Fontaine, Marijke M. Westra, Ilze Bot, Han Jin, Aimée J. P. M. Franssen, Martine Bot, Saskia C. A. de Jager, Ivan Dzhagalov, You-Wen He, Bart J. M. van Vlijmen, Marion J. J. Gijbels, Chris P. Reutelingsperger, Theo J. C. van Berkel, Judith C. Sluimer, Lieve Temmerman, Erik A. L. Biessen

**Affiliations:** 1 Experimental Vascular Pathology Group, Department of Pathology, Cardiovascular Research Institute Maastricht, Maastricht University Medical Center, Maastricht, the Netherlands; 2 Division of BioTherapeutics, Leiden Amsterdam Centre for Drug Research, Leiden University, Leiden, the Netherlands; 3 Laboratory for Experimental Cardiology, University Medical Center Utrecht, Utrecht, the Netherlands; 4 Institue of Microbiology and Immunology, National Yang-Ming University, Taipei, 112 Taiwan; 5 Einthoven Laboratory for Vascular and Regenerative Medicine, Leiden University Medical Center, Leiden, The Netherlands; 6 Department of Internal Medicine, Division of Thrombosis and Hemostasis, Leiden University Medical Center, Leiden, the Netherlands; 7 Department of Molecular Genetics, Cardiovascular Research Institute Maastricht, Maastricht University, Maastricht, the Netherlands; 8 Department of Biochemistry, Cardiovascular Research Institute Maastricht, Maastricht University, Maastricht, the Netherlands; 9 Centre for Cardiovascular Science, University of Edinburgh, Edinburgh, UK

**Keywords:** Apoptosis, Atherosclerosis

## Abstract

The anti-apoptotic protein myeloid cell leukemia 1 (Mcl-1) plays an important role in survival and differentiation of leukocytes, more specifically of neutrophils. Here, we investigated the impact of myeloid Mcl-1 deletion in atherosclerosis. Western type diet fed LDL receptor-deficient mice were transplanted with either wild-type (WT) or LysMCre Mcl-1^fl/fl^ (Mcl-1^−/−^) bone marrow. Mcl-1 myeloid deletion resulted in enhanced apoptosis and lipid accumulation in atherosclerotic plaques. *In vitro*, Mcl-1 deficient macrophages also showed increased lipid accumulation, resulting in increased sensitivity to lipid-induced cell death. However, plaque size, necrotic core and macrophage content were similar in Mcl-1^−/−^ compared to WT mice, most likely due to decreased circulating and plaque-residing neutrophils. Interestingly, Mcl-1^−/−^ peritoneal foam cells formed up to 45% more multinucleated giant cells (MGCs) *in vitro* compared to WT, which concurred with an increased MGC presence in atherosclerotic lesions of Mcl-1^−/−^ mice. Moreover, analysis of human unstable atherosclerotic lesions also revealed a significant inverse correlation between MGC lesion content and Mcl-1 gene expression, coinciding with the mouse data. Taken together, these findings suggest that myeloid Mcl-1 deletion leads to a more apoptotic, lipid and MGC-enriched phenotype. These potentially pro-atherogenic effects are however counteracted by neutropenia in circulation and plaque.

## Introduction

Antiapoptotic Mcl-1 is a member of the apoptosis regulating Bcl-2 family^1^. It directly interacts with pro-apoptotic BH3-only proteins Bim and Bid and multidomain proapoptotic Bad^2–4^, thereby inhibiting apoptosis. Mcl-1 is expressed in various tissues including hematopoietic cells^5^, in which its overexpression delays cell death in response to various stimuli^6^. Indeed Mcl-1 is critical for neutrophil survival^7–10^ at all differentiation stages^11^. Survival of macrophages lacking Mcl-1 does not seem to be impacted^9^, albeit that Mcl-1 deficient macrophages were observed to be more sensitive to apoptosis induced either by phagocytosis^10^, or infection^12^.

While monocytes and macrophages have been the subject of extensive studies in the atherosclerosis field for years^13^, neutrophils were only more recently investigated due to their scarce presence in atherosclerotic plaque. Nevertheless, it is now believed that neutrophils are instrumental in plaque development and later destabilization, as we and others have shown^14–16^. Warnatch *et al*. demonstrated that Neutrophil Extracellular Traps (NETs) prime macrophages to produce inflammatory cytokines, resulting in increased atherogenesis^17^. Dissection of the contribution of neutrophils to atherosclerotic plaque progression has however remained difficult, since other cell populations are also affected when neutrophils are experimentally manipulated. Deletion of Interferon Regulatory Factor 8 (IRF8) for example results in neutrophilia which exacerbates atherosclerosis in IRF8^−/−^ ApoE^−/−^ chimeric mice, but was at the same time accompanied by changes in monocytes and several dendritic cell populations^18,19^. In addition, neutrophils have thus far not been depleted during the complete course of atherosclerosis. Neutrophil depleting antibodies such as the anti–polymorphonuclear leukocyte (PMN) antibody cannot be sustained for more than 4 weeks in mice without affecting monocyte counts^16^.

As Mcl-1 is vital for neutrophil survival, whilst presumably having mild effects on macrophage apoptosis, we hypothesized that myeloid Mcl-1 deletion could serve as an efficient neutropenia model during the full pathogenesis of atherosclerosis. Moreover, although several Bcl-2 family members have been investigated in the context of atherosclerosis^20–23^, the role of Mcl-1 in disease progression has not been assessed thus far. To this end, we studied effects of specific deletion of Mcl-1 in the lysozyme M expressing myeloid subsets neutrophils and macrophages on early and advanced atherosclerosis using bone marrow transplantation of Mcl-1^fl/fl^ LysMCre or wild-type (WT) bone marrow into low density lipoprotein receptor-null (LDLr^−/−^) mice. Our study shows that myeloid Mcl-1 deletion indeed has a profound impact on neutrophil survival. However, Mcl-1 deficient macrophages were seen to accumulate more lipids *in vitro* and *in vivo* and were subsequently more sensitive to apoptosis. Moreover, we show that Mcl-1 is implicated in the fusion of macrophages. These combined effects however counteracted each other affecting only viable cell plaque composition, but not plaque growth.

## Results

### Mcl-1 deletion altered neutrophil levels and characteristics

**Figure 1:**
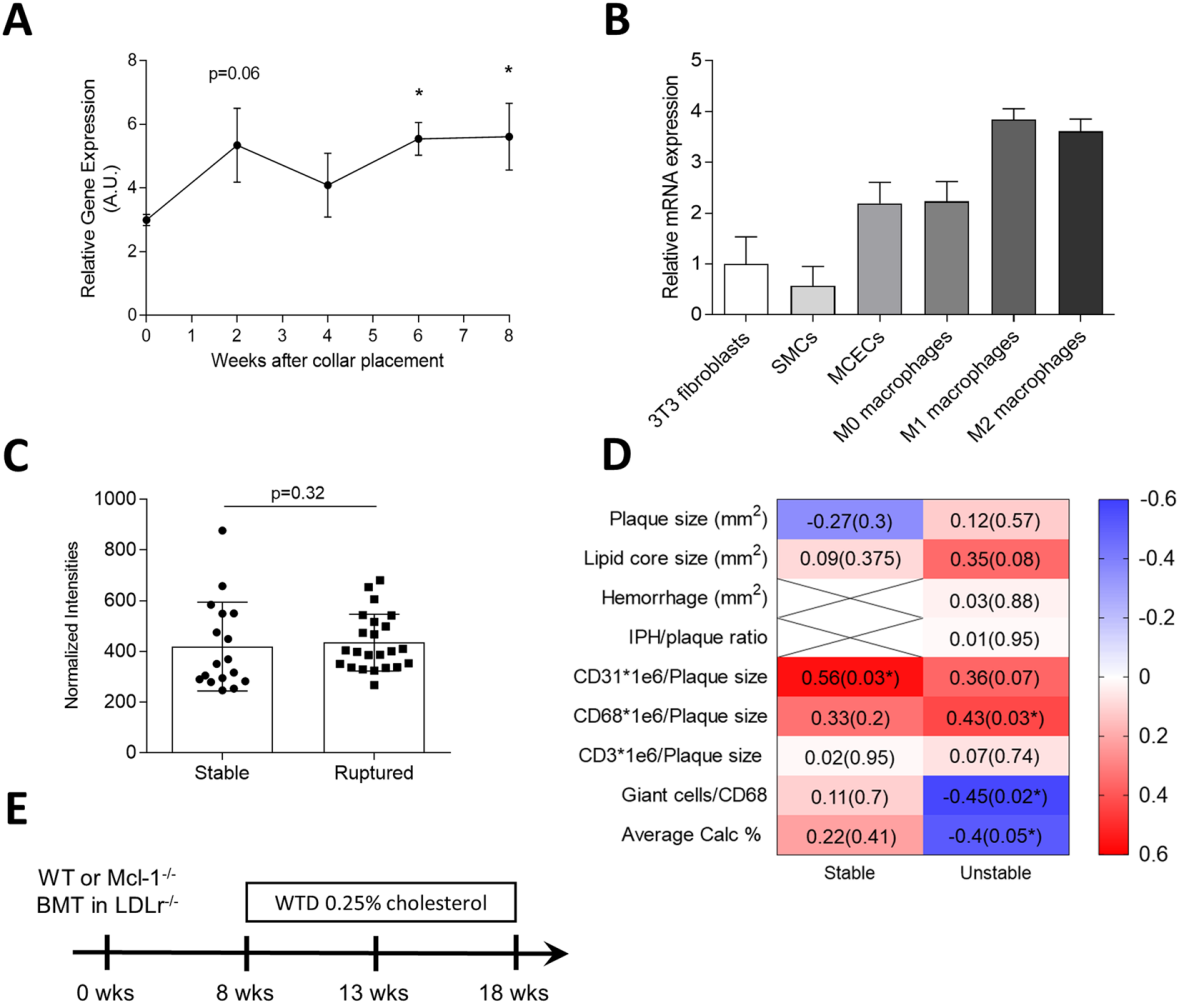
Regulation of Mcl-1 expression in atherosclerosis. (**A–C**) Mcl-1 gene expression measured by RT-qPCR. (**A**) Vascular Mcl-1 expression corrected for HPRT housekeeping gene in a model of collar induced carotid artery atherogenesis in LDLr^−/−^ mice. (**B**) Mcl-1 expression corrected for 18 S housekeeping gene in different mouse cell types. SMCs: smooth muscle cells, MCECs: mouse cardiac endothelial cells. (**C**) Mcl-1 gene expression in human atherosclerotic plaques, represented by microarray normalized intensities. (**D**) Heatmap showing Pearson correlation coefficient (p-values) of Mcl-1 human plaque gene expression correlation with clinical plaque traits. N = 22/23 (stable/unstable). (**E**) Lethally irradiated LDLr^−/−^ mice were reconstituted with WT or Mcl-1^−/−^ bone marrow, and after 8 weeks of recovery, put on a Western Type Diet (WTD) containing 0.25% cholesterol for 5 weeks (n = 17) or 10 weeks (n = 19). All data is presented as mean ± SEM. *p < 0.05.

**Figure 2:**
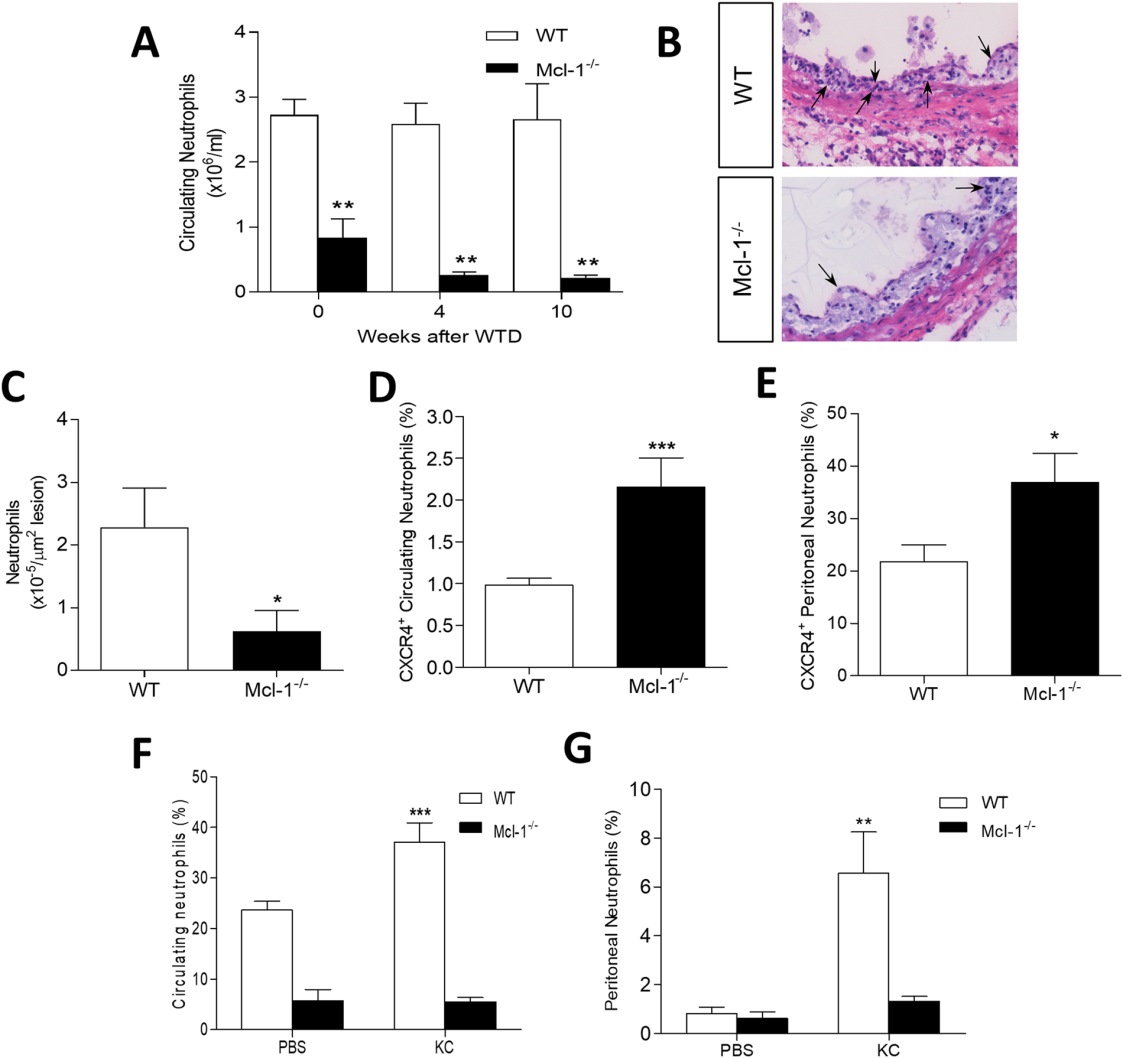
Mcl-1^−/−^ chimeric mice have altered neutrophil levels and characteristics. (**A**) Circulating neutrophils were defined as Gr1+ and measured by flow cytometry in blood samples obtained from tail vein of WT and Mcl-1^−/−^ bone marrow chimeras. (**B**) Representative H&E pictures of WT and Mcl-1^−/−^ atherosclerotic plaques (n = 19). Neutrophils are indicated by the arrow. (**C**) in aortic root atherosclerotic lesions of BM transplanted LDLr^−/−^ after 10 weeks of WTD (n = 19). (**D**,**E**) CXCR4 expression in circulating and peritoneal neutrophils respectively assessed by flow cytometry (CXCR4 positive cells within the neutrophil gate of **A**). (**F,G**) Circulating and peritoneal neutrophil levels respectively 2 h after CXCL1 injection measured by flow cytometry (CXCR4 positive cells within the neutrophil gate of **A**). Data is presented as mean ± SEM. ***p < 0.001, **p < 0.01 and *p < 0.05.

We first quantified Mcl-1 gene expression during atherogenesis. Mcl-1 levels in collar-induced carotid artery lesions of LDLr^−/−^ mice gradually increased during lesion development and in particular in advanced plaques, six weeks after collar induction (Fig. 1A). Of note, this increase was not validated at the protein level. Mcl-1 was mostly expressed in activated macrophages (M1- or M2-macropahges), as compared to other cell types (Fig. 1B). Mcl-1 was also detectable in human atherosclerotic plaques, and its expression did not differ between stable and unstable plaque (Fig. 1C). However, Pearson correlation analysis revealed that Mcl-1 expression did correlate with pathogenic plaque traits, with more traits in unstable plaques, suggesting an involvement in the disease process (Fig. 1D). To verify whether Mcl-1 deletion indeed resulted in an efficient neutropenia model in the context of atherosclerosis, Ldlr^−/−^ recipient mice were transplanted with LysMCre Mcl-1^fl/fl^ (hereafter Mcl-1^−/−^) or wild type (WT) bone marrow (Fig. 1E). Mcl-1^−/−^ mice showed similar serum cholesterol and triglyceride levels, and body weight (see Supplementary Data Fig. IA–C). Compatible with the notion that Mcl-1 is essential for neutrophil survival^7^, circulating and splenic neutrophil numbers were sharply reduced by 80% and 86%, respectively in Mcl-1^fl/fl^ LysMcre mice^9^. Circulating neutrophils were depressed in Mcl-1^−/−^ chimeras both at baseline (82% depletion) and even more so under hyperlipidemic conditions (91% depletion) (Fig. 2A). Likewise, neutrophil content in Mcl-1^−/−^ atherosclerotic lesions was decreased, albeit to a lower extent than in blood (Fig. 2B,C), hinting to an enhanced adhesive capacity or faster turnover of residual neutrophils in circulation. Considering that an elevated CXCR4/CXCR2 balance is associated with regress to the bone marrow and that CXCR4 is an established measure of neutrophil ageing^16^, we examined neutrophil phenotype. CXCR4 expression on circulating and peritoneal residual neutrophils was increased (Fig. 2D,E, respectively), suggesting hyperactivation and increased SDF1 migratory capacity. Moreover, responsiveness of remaining neutrophils to the potent neutrophil chemokine CXCL1 was blunted, concordant with the reduced CXCR2 expression by pre-apoptotic neutrophils^16^. Peritoneal neutrophil influx 2 hours after i.p. injection of CXCL1 was prominent in WT transplanted mice, whereas Mcl-1^−/−^ transplanted mice only showed a minor, non-significant, increase in peritoneal neutrophils (Fig. 2F,G). Of note, neutrophil recruitment was paralleled by stromal egress of neutrophils into circulation in WT, but not Mcl-1^−/−^ mice (data not shown). Taken together, these results confirm Mcl-1 as a crucial neutrophil survival factor, also under hyperlipidemic conditions, and demonstrate that Mcl-1 myeloid deletion can be used as a genetic tool to induce a long-lasting, severe neutropenia in atherosclerosis.

### Myeloid Mcl-1 deletion increased plaque apoptotic cell content but did not affect atherosclerotic lesion size

**Figure 3:**
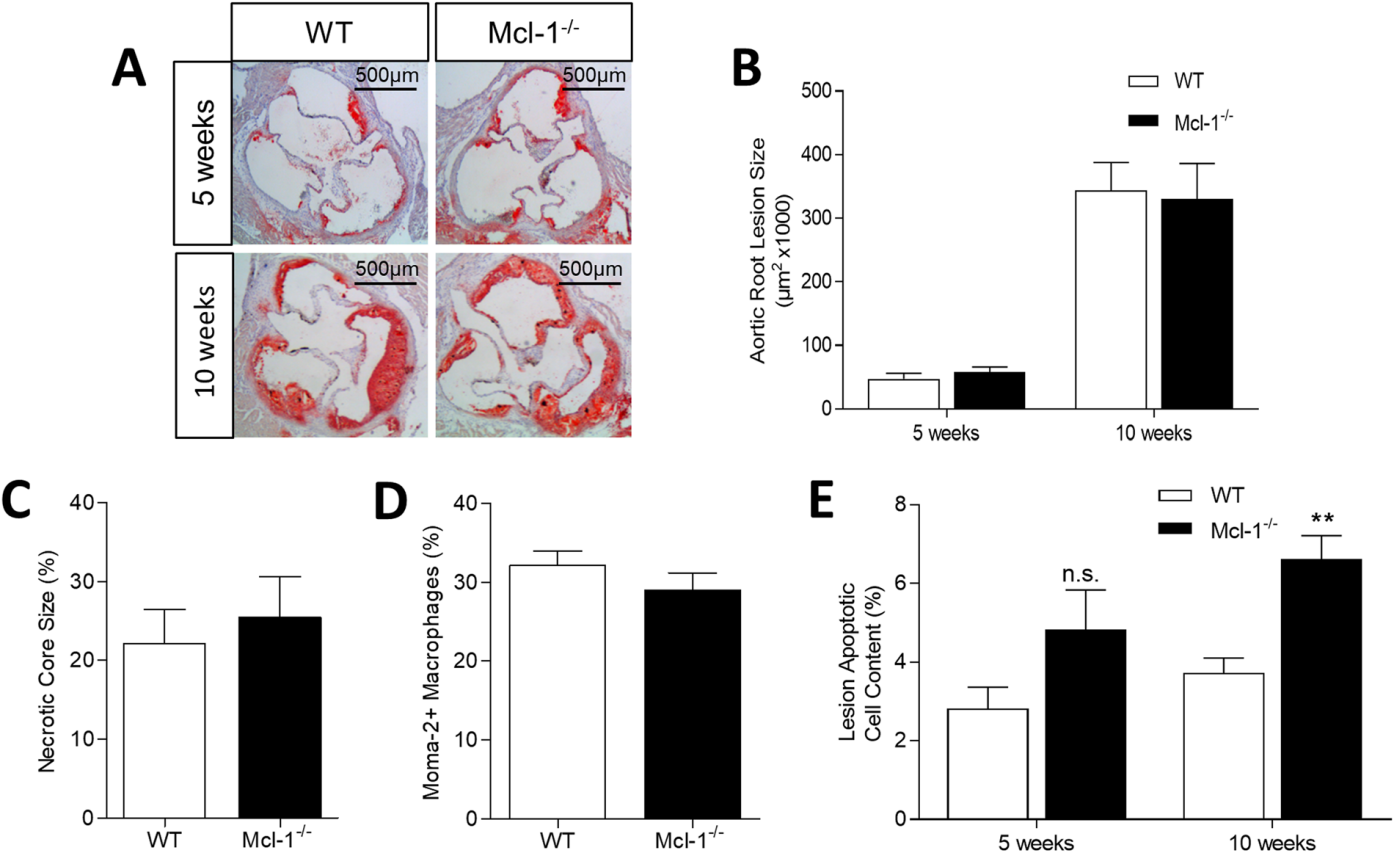
Effect of myeloid Mcl-1 deficiency on atherosclerotic lesion size and composition. (**A**) Representative micrographs of Oil Red O stained aortic root sections in WT and Mcl1^−/−^ mice after 5 and 10 weeks of WTD. (**B**) Atherosclerotic lesion size after 5 weeks or 10 weeks of WTD quantified on Oil Red O staining using Leica image analysis system. (**C**) Plaque necrotic core size after 10 weeks of WTD. (**D**) Plaque macrophage content after 10 weeks of WTD assessed on Moma-2 positive staining. (**E**) Plaque apoptotic cell content after 5 weeks or 10 weeks of WTD, measured by TUNEL staining. Data is presented as mean ± SEM. **p < 0.01.

Despite its profound effects on circulating neutrophils, and on plaque neutrophil content, myeloid Mcl-1 deletion did neither alter early nor advanced plaque area, necrotic core size, or plaque macrophage content as compared to controls (Fig. 3A–D). We did observe an increase in plaque apoptosis by 71% and 77% in atherosclerotic lesions of Mcl1^−/−^ mice fed a WTD for 5 and 10 weeks, respectively, compared to WT mice (Fig. 3E), suggesting that Mcl-1 not only plays an important role in the survival of neutrophils, but also of other myeloid plaque-resident cells, such as plaque macrophages and foam cells.

### Mcl-1 myeloid deletion enhanced lipid-induced apoptosis sensitivity and lipid loading capacity of macrophages

**Figure 4:**
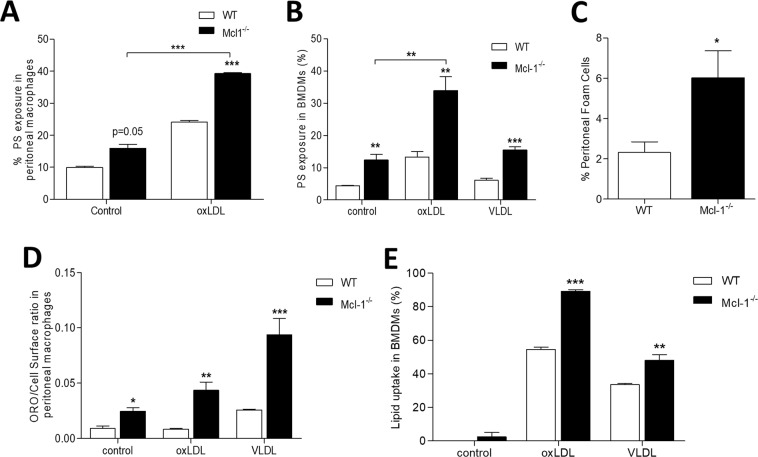
Effect of Mcl-1 myeloid deletion on macrophage apoptosis and lipid loading. (**A**) PS exposure of unstimulated or oxLDL (40 µg/ml) stimulated peritoneal macrophages measured by Annexin-V-OG staining. (**B**) PS exposure of unstimulated or oxLDL/VLDL stimulated bone marrow derived macrophages (BMDMs) measured by Annexin-V-OG staining. (**C**) Peritoneal foam cell presence of WT and Mcl-1^−/−^ mice after 10 weeks of WTD assessed by Oil Red O staining *in vitro*. (**D**) Lipid loading capacity of peritoneal macrophages after oxLDL (20 µg/ml) and vLDL (50 µg/ml) exposure. (**E**) Lipid uptake capacity of BMDMs after oxLDL (20 µg/ml) and VLDL (50 µg/ml) incubation. Data is presented as mean ± SEM. ***p < 0.001, **p < 0.01 and *p < 0.05.

We next investigated the role of Mcl-1 in macrophages and foam cells in atherosclerosis, reasoning that hyperlipidemia could unleash a role for Mcl-1 in plaque macrophage biology, as has been described for macrophages undergoing infection^12^. Mcl-1^−/−^ peritoneal macrophages displayed a higher level of apoptosis already at baseline, confirming that the hyperlipidemic environment by itself triggered the Mcl-1 survival program (Fig. 4A). Moreover, Mcl-1^−/−^ peritoneal macrophages, as well as Mcl-1^−/−^ BMDMs (see Supplementary Data Fig. IIA,B), displayed an increased sensitivity towards oxLDL-induced cell death compared to WT macrophages (Fig. 4A-B and Supplementary Data Fig. IIIA–E). Interestingly, peritoneal foam cell numbers were increased by 2.5-fold in Mcl-1^−/−^ compared to WT transplanted mice (Fig. 4C), whereas total peritoneal macrophage numbers were unchanged (data not shown). This finding led us to examine the lipid loading capacity of peritoneal macrophages *in vitro*. In agreement with the elevated peritoneal foam cell counts *in vivo*, lipid accumulation in non-stimulated Mcl-1^−/−^ BMDMs *in vitro* was markedly increased (Fig. 4D). Lipid loading in WT macrophages remained unchanged after incubation with oxLDL for 24 h but was substantially enhanced in Mcl-1^−/−^ macrophages (Fig. 4D). While incubation with VLDL increased lipid content in both WT and Mcl-1^−/−^ macrophages, this increase was considerably higher in the latter cells (Fig. 4D). In keeping with what is observed in peritoneal macrophages, Mcl-1^−/−^ BMDMs also showed an increased lipid uptake capacity (Fig. 4E). Thus, under conditions of hyperlipidemia Mcl-1 appears to be an active regulator of macrophage survival as well as of macrophage lipid loading.

### Mcl1 deletion induces multinucleated giant cell formation

**Figure 5:**
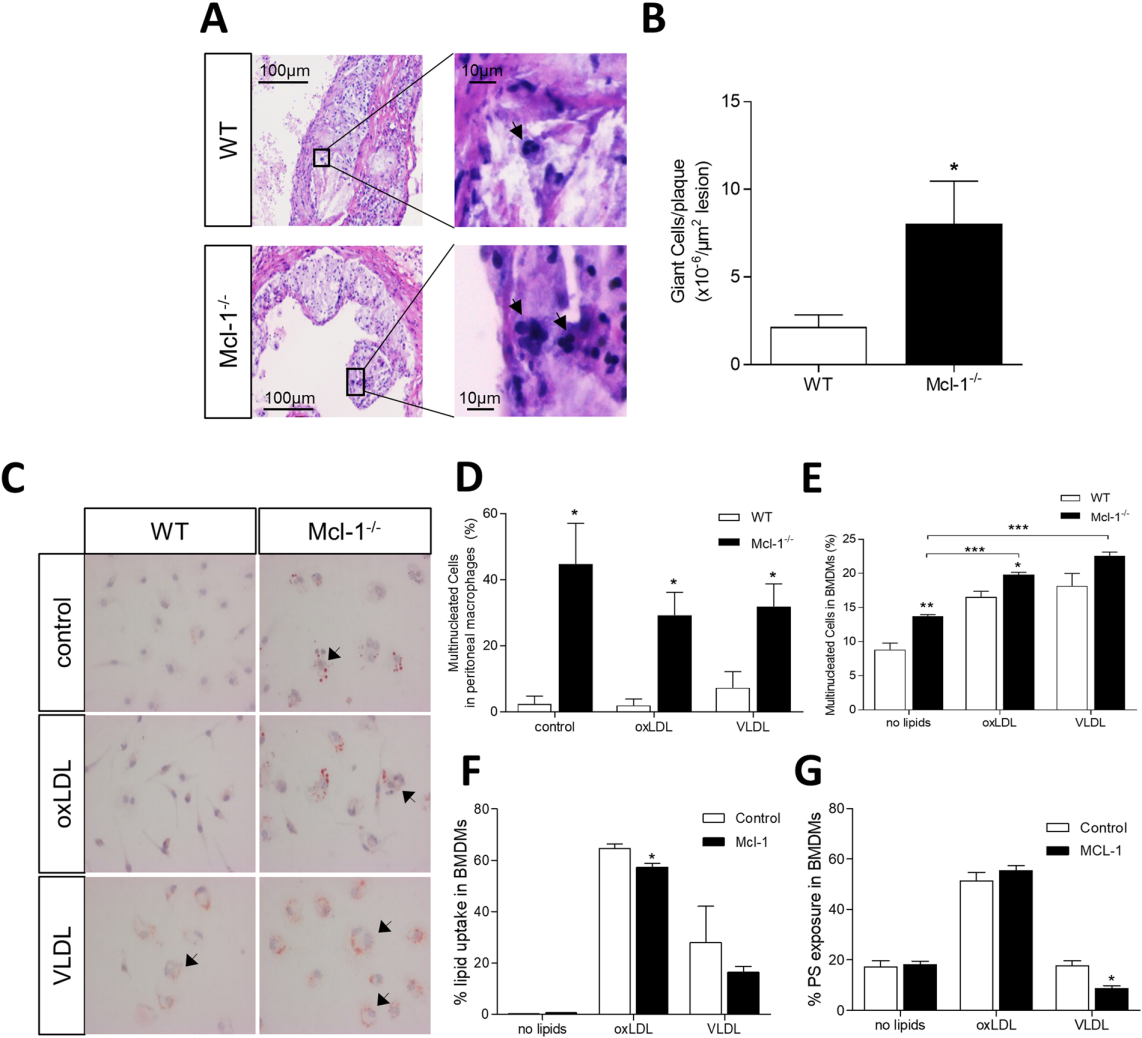
Effect of Mcl-1 deletion on macrophage fusion. (**A**) Representative pictures of mouse aortic root sections stained with H&E. Multinucleated Giant Cells are indicated by the arrow. MGCs were quantified as cells containing 2 or more round nuclei. (**B**) MGCs quantification in the atherosclerotic lesions of WT and Mcl1^−/−^ chimeras after 10 weeks of WTD. (**C**) Representative pictures of Oil Red O stained unstimulated, oxLDL or VLDL stimulated peritoneal macrophages. Multinucleated Giant Cells are indicated by the arrow (**D**) Quantification of Giant Cell population in peritoneal macrophages depicted in (**C**). (**E**) MGCs in BMDMs after 6d of culture in the presence of either oxLDL, VLDL or nothing. (**F**) Lipid loading capacity of MGCs after 13 d in culture. (**G**) PS exposure in MGC population after 13 d in culture. Data is presented as mean ± SEM. ***p < 0.001, **p < 0.01 and *p < 0.05.

**Figure 6:**
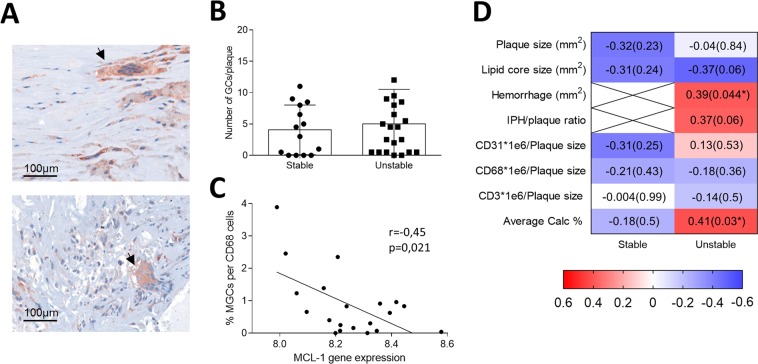
Presence of Multinucleated Giant Cells in human atherosclerotic plaques. (**A**) Representative pictures of cathepsin K stained human unstable plaques. Multinucleated Giant Cells are indicated by the arrow (**B**) MGCs are quantified as cells positive for cathepsin K and containing 2 or more round nuclei. (**C**) Pearson correlation analysis showing coefficient (p-values) between MGCs presence and MCL-1 gene expression levels in human unstable plaque segments (n = 18). (**D**) Heatmap showing Pearson’s correlation coefficient/p-values between the presence of MGCs and other clinical plaque traits. N = 22/23 (stable/unstable). *Indicates significant correlation.

Much to our surprise, we noticed the abundant presence of multinucleated giant cells (MGCs) in plaques of Mcl-1^−/−^ transplanted animals after 10 weeks on WTD. Indeed, MGC content per plaque was increased by 118% in the Mcl-1^−/−^ transplanted animals as compared to WT (Fig. 5A,B), suggesting that Mcl-1 myeloid deficiency leads to the formation of MGCs. In addition, Mcl-1 myeloid deficiency increased MGCs formation by peritoneal macrophages *in vitro* both in control conditions and after incubation with oxLDL and VLDL (Fig. 5C,D). To further investigate the Mcl-1-dependent changes underlying MGC formation, we incubated WT BMDMs with IL4 and GM-CSF, two known cytokines to induce giant cells^24,25^, and compared them to Mcl-1^−/−^ BMDMs. While giant cell formation was unaffected after one week of culture (data not shown), it was significantly increased in Mcl-1^−/−^ BMDMs compared to WT BMDMs at 13 days of culture (P value = 0.002, Fig. 5E). Mechanistically, the effect was partially mediated by hyperlipidemia, as oxLDL or VLDL increased MGC formation in BMDM-derived cells to a larger extent (Fig. 5E). Of note, after 13 days in culture, Mcl-1^−/−^ BMDMs had partially lost their increased capacity of lipid uptake and subsequent lipid-induced apoptosis (Fig. 5F,G, respectively). To assess the relevance of Mcl-1-dependent MGC formation in human atherosclerosis, we quantified MGCs in human plaques. Cathepsin K^+^ multinucleated cells were frequently found in both stable and unstable plaques (Fig. 6A,B). Interestingly, their presence inversely correlated with Mcl-1 gene expression in unstable plaque, and only in unstable lesions associated significantly with lesion hemorrhages and calcifications (P value = 0.044 and 0.055, respectively, Fig. 6C,D). Thus, our results unveil a hitherto unknown link between Mcl-1 and MGC formation, which is influenced by hyperlipidemia, an association potentially preserved in human atherosclerotic lesions as well.

## Discussion

In this study, we evaluated the effects of myeloid Mcl-1 deletion and its accompanying neutropenia on atherosclerosis progression. In addition to extreme neutropenia, Mcl-1 deficiency resulted in increased macrophage apoptosis and lipid handling, and triggered multinucleated giant cell formation. First, we found that myeloid Mcl-1 deletion dramatically reduced neutrophil numbers both in circulation and in atherosclerotic lesions. This is in keeping with extensive data on the vital role of Mcl-1 in neutrophil survival^9,10,26,27^ which as we now demonstrated remains valid in a hyperlipidemic environment. On the other hand, Mcl-1 overexpression^28,29^ and Bim deletion^23^, a pro-apoptotic Bcl-2 family member and Mcl-1 antagonist, do not affect circulating myeloid cell numbers, suggesting that physiological Mcl-1 levels are sufficient for normal cell function. Our work thus provides a mouse model for continuous neutropenia, an important advantage to models used in earlier studies addressing neutrophil contribution to atherogenesis. Though Zernecke *et al*. demonstrated that CXCR4 blockade aggravated atherosclerosis due to increased neutrophil recruitment to the plaque, they were unable to extend the neutrophil depletion beyond a 4 week period^16^. They did show that plaques of neutrophil depleted mice were smaller and had a lower neutrophil and macrophage content, but did not evaluate plaque apoptosis or necrotic core size. Similarly so, CCL3^−/−^ LDLr^−/−^ bone marrow chimeras with 50% less neutrophils, developed smaller atherosclerotic lesions^30^. Consequently, we were highly surprised by the unaffected atherosclerotic lesion burden in neutropenic LysMCre Mcl-1^−/−^ mice.

Apparently the atheroprotective effects of Mcl-1 deletion in neutrophils in LDLr^−/−^ mice are counteracted by potentially pro-atherogenic effects on other cell types targeted by the LysM conditional Mcl-1 deletion, we therefore examined in greater detail the role of Mcl-1 in atherosclerotic LysM^+^ monocytes and macrophages.

Although Mcl-1 loss was previously shown to have no effect on monocyte and macrophage development in wild type mice^9,10^, Mcl-1^−/−^ macrophages were more sensitive to apoptosis upon an infection^12^ or phagocytic challenge^10^. We found that the apoptotic cell content in advanced aortic root lesions (10 weeks of WTD) was increased by 44% in mice with myeloid Mcl-1 deficiency. As neutrophils are only scarcely present in advanced lesions^14^ and most apoptotic cells were located in the central atheroma (data not shown), the high apoptotic cell density is likely to reflect dying LysM^+^ plaque macrophages. Our work thus identifies Mcl-1 as a major survival protein in atherosclerotic lesions. Atherosclerotic lesion burden was however unaltered in Mcl-1^−/−^ BM recipients, as were necrotic core, macrophage and collagen content. Similar results were obtained when studying plaque initiation five weeks after WTD. Our results correspond with those from Thorp *et al*.^21^, who showed increased macrophage apoptosis, but unchanged lesion burden in Bcl-2^flox^-LysMCre ApoE^−/−^ mice that are deficient in macrophage and neutrophil Bcl-2^21^. In turn, hematopoietic Bim deficiency, a pro-apoptotic Bcl-2-family member, had no impact on macrophage apoptosis and lesion burden in LDLr^−/−^ mice^23^. Thus, Mcl-1^−/−^ deletion in macrophages led to higher apoptosis level in advanced plaques, however it is clear from the above that this not always translates into a pro-atherogenic plaque progression.

In addition to an increased sensitivity to oxLDL induced cell death, Mcl-1^−/−^ macrophages showed augmented lipid accumulation after incubation with oxLDL and VLDL. In keeping, we observed elevated foam cell levels *in vivo* in the peritoneal cavity of Mcl-1^−/−^ BM compared to WT BM recipients. These findings seem to contrast with those of Halvorsen *et al*.^31^, who reported reduced IL-10 induced oxLDL loading by THP-1 macrophages *in vitro* after siRNA mediated silencing of Mcl-1 and Bfl-1 expression. The authors did not assess effects of Mcl-1 inhibition alone, without IL-10 stimulation. Based on our data we hypothesize that the apoptosis-prone phenotype of Mcl-1^−/−^ macrophages is at least partly caused by the increased uptake of lipids.

Another remarkable characteristic was the high propensity of Mcl-1^−/−^ cells to form multinucleated giant cells (MGCs). MGCs, a hallmark of several chronic inflammatory diseases^32,33^, originate from monocyte-macrophage lineage and result from cell fusion^32,34^. Giao *et al*. have illustrated the presence of TRAP-positive (osteoclast like giant) cells in close relation to calcified regions and TRAP-negative MGCs in advanced human atherosclerotic plaques^35^. In addition, Samokhin *et al*.^36^ showed that mice fed a Paigen diet displayed a 4-fold increase in MGC number in atherosclerotic lesions^36^. In our study, we observed a higher macrophage fusion capacity in both Mcl-1^−/−^ peritoneal and BMDMs, which increased even more upon oxLDL or VLDL stimulation. Furthermore, Mcl-1^−/−^ deficiency in BMDMs seemed to promote MGC formation, independently of lipid uptake. In line with our *in vitro* findings, Mcl-1^−/−^ atherosclerotic plaques had a 4-fold increase in MGC presence as compared to WT lesions. Additionally, the presence of MGCs in human unstable plaques correlated negatively with Mcl-1 gene expression and significantly associated with hemorrhages and calcifications in the lesion. The exact role of MGCs in atherosclerosis is not yet fully understood, however it was previously shown that MGCs facilitate vascular smooth muscle cell migration in the context of atherosclerosis by producing cathepsin K and destroying the elastin fibers^36^. Although not providing conclusive evidence, our findings support such pro-atherogenic role of MGCs. To our knowledge, we are the first to implicate Mcl-1 in the fusion of macrophages. Possibly, this is related to increased oxidative phosphorylation capacity for energy production in Mcl-1^−/−^ deficient BMDMs (data not shown), however the exact mechanism by which Mcl-1 induces MGC formation remains to be investigated.

In summary, myeloid Mcl-1 deficiency led to a profound and sustained neutropenia in hyperlipidemic LDLr^−/−^ mice accompanied by enhanced oxLDL induced macrophage death *in vitro*, as well as increased atherosclerotic lesion apoptosis. Furthermore, Mcl-1 deficiency was shown to enhance lipid uptake and induce the formation of MGCs *in vitro* and *in vivo*. In line with the observation in mice, Mcl-1 gene expression negatively correlated with the presence of MGCs in human unstable plaque. Taken together, the markedly lower neutrophil numbers mask the combined effects of a more lipid rich and apoptotic plaque in myeloid Mcl-1 deficient animals, and result in an unchanged lesion development and progression. Our results clearly identify Mcl-1 as a macrophage survival protein under hyperlipidemia and uncover a hitherto unknown role for Mcl-1 in MGC formation.

## Material and Methods

### Animals

All animal work was approved by animal regulatory authority of Leiden University and performed in compliance with Dutch national guidelines. Low density lipoprotein receptor-null (LDLr^−/−^) mice were obtained from the local animal breeding facility. Mcl-1^fl/fl^ LysMcre mice were obtained from the Department of Immunology, Duke University Medical Center, USA and were backcrossed to C57BL/6J.

### Mcl-1 gene expression during atherogenesis

Twenty male LDLr^−/−^ mice were fed a Western-type diet (WTD) two weeks prior to surgery and throughout the experiment. Atherosclerotic carotid artery lesions were induced by perivascular collar placement^37^. Carotid Mcl-1 gene expression was analyzed prior to and two, four, six and eight weeks after collar placement (n = 4, per timepoint).The mice were anaesthetized and perfused with phosphate buffered saline (PBS) after which both common carotid arteries were isolated. After dissection of the adventitia, plaque containing segments were excised based on macroscopic examination, snapfrozen and stored at −80°c. Two to three atherosclerotic plaques were pooled per sample and total RNA was isolated using Trizol reagent (Invitrogen). Gene expression was analyzed by real time PCR (qPCR) using ABI PRISM 7700 Sequence Detector (Applied Biosystems) with SYBR-Green technology. *Mcl-1* and housekeeping gene *Hprt* primers used are listed in Supplementary Table 1.

### Mcl-1 gene expression in different cell types

Murine 3T3 fibroblasts, smooth muscle cells (SMCs), cardiac endothelial cells (MCECs), and BMDMs stimulated with either lipopolysaccharide (10 ng/ml) and Interferon-γ (100 U/ml) (M1 macrophage) or Interleukin-4 (20 ng/ml) (M2 macrophage) were cultured. Total RNA was extracted and gene expression was analyzed by qPCR. *Mcl-1* and housekeeping gene *18 S* primers used are listed in Supplementary Table 1.

### Bone marrow transplantation and atherosclerosis induction

Male recipient LDLr^−/−^ mice were housed in sterile ventilated cages with food (RM3, Special Diet Services) and water ad libitum. Antibiotics (83 mg/l ciprofloxacin, 67 mg/l Polymixin B and 5 g/l sugar) were supplied in the drinking water. Mice were exposed to a single dose of 9 Gy total body irradiation (0.19 Gy/min, 200 kV, 4 mA) using an Andrex Smart 225 Röntgen source (YXLON International) one day before transplantation. Bone marrow was extracted from femur and tibia of male Mcl-1^fl/fl^ LysMcre (hereafter Mcl-1^−/−^) donors and wild type (WT) littermates. Irradiated LDLr^−/−^ mice received 2.5 × 10^6^ BM cells of either Mcl-1^−/−^ (n = 17) or WT (n = 15) via tail vein injection. After a recovery period of eight weeks mice were put on a WTD containing 0.25% cholesterol and 15% cacao butter (Diet W, Special Diet Services) for an additional five (plaque initiation) or ten weeks (advanced plaque).

### Blood cell analysis and flow cytometry

Blood samples were taken by tail bleeding immediately before BMT, prior to (week 0) and after four weeks of WTD feeding (week 4) and at the time of sacrifice (week 5 or week 10). Peritoneal leukocytes were isolated at the time of sacrifice by peritoneal lavage with 10 ml PBS. Whole blood and peritoneal lavage samples were analyzed using a Sysmex blood cell analyzer (XT-2000i). For flow cytometry, WBC and peritoneal leukocytes were stained with fluorescently labelled antibodies against F4/80, CD19, CD4, CD71 and CD11b (eBioscience) and Gr1, CD8 and CXCR4 (BD Pharmingen). Fluorescence-activated cell sorting (FACS) analysis was performed on a FACSCalibur with CellQuest software (BD Biosciences).

### Tissue harvesting and analysis

Two hours before sacrifice, Mcl-1^−/−^ or WT mice (n = 5) received intraperitoneal injections of CXCL1 (200 ng/ml in 1 ml PBS) or PBS control. The mice were anesthetized and perfused with PBS. Cryosections of the aortic root tissue were stained with hematoxylin and eosin (HE) or Oil Red O. Lesion size was quantified using a Leica DMRE microscope with camera and Leica Qwin Imaging software (Leica Ltd). MGCs were defined as macrophages with two or more round nuclei on the HE slides and quantified by an animal pathologist. Immunohistochemical stainings were performed for macrophage (MOMA-2, Sigma) and vSMC (α-smooth muscle actin, Sigma) content. Apoptotic cell content was quantified using terminal deoxytransferase dUTP nick-end labeling (TUNEL) kit (Roche Diagnostics).

### LDL and VLDL isolation and oxLDL preparation

LDL and very low-density lipoprotein (VLDL) were isolated from human plasma by density gradient ultracentrifugation for 20 h at 4 °C^38^. LDL concentration was adjusted to 0.5 mg/ml with PBS and oxidized by incubation with 0.32 mM CuSO_4_ overnight at 37 °C after which the oxidation reaction was terminated by addition of 50 µM EDTA. oxLDL was then dialyzed against PBS containing 10 µM EDTA for 24 h. Final oxLDL and VLDL concentration were measured using the bicinchonic acid protein kit (Thermo Fisher Scientific).

### BMDMs and peritoneal macrophages

Bone marrow cells were isolated by flushing femurs and tibia with PBS and single cell suspensions were obtained by passing the suspension through a 70 µm nylon cell strainer (BD Falcon). Bone marrow cells were differentiated into macrophages by culturing in RPMI-1640 medium (GIBCO Invitrogen), supplemented with 10% (vol/vol) heat inactivated fetal calf serum (FCS) (GIBCO, Invitrogen), glutamine (2 mM), penicillin (100 U/ml), streptomycin (100 μg/ml) and 15% (vol/vol) L929-conditioned medium for 7 days. After differentiation, BMDMs were harvested with lidocaine and replated at 0.5 × 10^5^ cells/well in a 96 well imaging plate (BD #353219, Corning Life Sciences) for apoptosis, lipid uptake and fusion assay. Peritoneal leukocytes were isolated from Mcl-1^−/−^ and WT mice following an i.p. injection of PBS before sacrifice. Cells were pooled from 2–3 mice per genotype and plated at 0.25 × 10^6^ cells/well in 8 chamber culture slides or 24-well plates (BD Falcon) and non-adherent peritoneal macrophages were removed.

### Lipid loading of BMDMs and peritoneal macrophages

Adherent peritoneal macrophages were stimulated with 20 µg/ml oxLDL or 50 µg/ml VLDL for 24 hours, after which slides were washed with PBS and stained with Oil Red O (ORO). Lipid loading was quantified as the ratio between the ORO stained area and total cell surface. BMDMs were loaded with 20 µg/ml oxLDL or 50 µg/ml VLDL combined with 5 µg/ml and 12.5 µg/ml Topfluor cholesterol (Avanti Polar Lipids) respectively for 2.5 h (D0) and 120 h (D5). Cell nuclei were stained with Hoechst (Sigma Aldrich) at 15 µg/ml.

### Apoptosis of peritoneal macrophages and BMDMs

Adherent peritoneal macrophages were stimulated with 40 µg/ml oxLDL for 24 hours. The macrophages were detached with Accutase (PAA Laboratories GmbH), stained with FITC-labeled Annexin-V (ImmunoTools) and propidium iodide (Sigma Aldrich) and subsequently analyzed by flow cytometry (FACSCalibur, BD Biosciences). For the BMDM apoptosis assay, cells were loaded with 20 µg/ml oxLDL or 50 µg/ml VLDL for 6 h (D0) or 120 h (D5) and subsequently stained with Hoechst and Oregon-green or Alexa-647 labelled Annexin-V for 15 minutes^39^.

### Fusion assay

BMDMs were loaded with 20 µg/ml oxLDL or 50 µg/ml VLDL for either 24 h (D1) or 120 h (D5). Macrophage fusion was measured by co-staining with calcein-AM (ThermoFisher Scientific) at 1 µg/ml and Hoechst. MGCs were defined as having two or more nuclei per calcein-segmented cell.

### Image analysis

For all 96-well plate immunofluorescence assays (apoptosis, lipid loading and cell fusion), nine images per well were taken using the high content analyzer (HCA) BD pathway 855 (BD Biosciences) with 10x objective and further analyzed using Attovision and DIVA software (BD Biosciences).

### Human atherosclerotic plaque collection

Stable and unstable human carotid atherosclerotic plaques segments (classified according to Virmani *et al*.^40^) were collected from the same symptomatic patient (n = 22/23) undergoing carotid endarterectomy in Maastricht University Medical Centre (MUMC, The Netherlands) and Zuyderland Medical Center (Sittard, the Netherlands). Collection, storage, and use in the Maastricht Pathology Tissue Collection (MPTC) were approved by medical ethical committee (16-4-181) and in accordance with the “Code for Proper Secondary Use of Human Tissue in the Netherlands” (http://www.fmwv.nl). Atherosclerotic plaque segments were alternatively snapfrozen for RNA and microarray analysis, or formalin-fixed for paraffin-embedding.

### Human atherosclerotic plaque histology

The human plaque sections adjacent to the snapfrozen segment were classified to determine plaque type according to Virmani *et al*.^40^. HE staining was used to quantify plaque size, lipid core size and hemorrhage. Alizarin red staining was done to measure the percentage of calcification.

### Human atherosclerotic plaque immunohistochemistry

All stainings were performed on adjacent plaque sections for vascular endothelial marker CD31 (Dako), macrophage marker CD68 (Dako), T-cell marker CD3 (Dako) and MGC marker (cathepsin K^41^). MGCs were defined as cathepsin-K positive cells having 2 or more round nuclei. MGC were quantified in all sections and averaged per patient. Relative abundance of MGC was calculated by dividing the number of MGC by that of CD68 positive cells.

### Human atherosclerotic plaque RNA extraction and transcriptomics

RNA was isolated by Guanidium Thiocyanate lysis followed by Cesium Chloride gradient centrifugation, and then purified using the Nucleospin RNAII kit. 750 ng of biotinylated cRNA per sample was hybridized to Illumina Human Sentrix-8 V2.0 BeadChip® and washed according to the Illumina standard procedure. Scanning was performed on the Illumina BeadStation 500. Raw expression data were extracted from the images using default settings and without normalization.

### Statistics

Values are expressed as mean ± standard error of the mean (SEM). All statistical analyses were performed using Prism (GraphPad Software). Statistically significant differences (p < 0.05) were evaluated using the Student’s t-test unless stated otherwise. Pearson correlation analysis was performed to assess the association between Mcl-1 gene expression or MGCs presence and clinical plaque traits. *p < 0.05, **p < 0.01 and ***p < 0.001.

## Supplementary information

Supplementary material

## Data Availability

All data measured and analysed during this study will be made available upon request.
