# Supplementary material for: Low human and murine Mcl-1 expression leads to a pro-apoptotic plaque phenotype enriched in giant-cells
Source: Sci Rep. 2019 Oct 10;9:14547. doi: 10.1038/s41598-019-51020-3 (PMC6787218; doi:10.1038/s41598-019-51020-3)
Supplement: Supplementary file 1 — Supplementary material [file 41598_2019_51020_MOESM1_ESM.pdf]

Low human and murine Mcl-1 expression leads to a pro-apoptotic plaque phenotype enriched in giant-cells.

Margaux A C Fontaine[1†], Marijke M Westra[2†], Ilze Bot[2], Han Jin[1], Aimée J P M Franssen[1], Martine Bot[2], Saskia C A de Jager[2,3], Ivan Dzhagalov[4], You-Wen He[4], Bart J M van Vlijmen[5,6], Marion J J Gijbels[1,7], Chris P Reutelingsperger[8],Theo J C van Berkel[2], Judith C Sluimer[1,9], Lieve Temmerman[1,*], Erik A L Biessen[1]

[†]These authors contributed equally

[*]Corresponding author (lieve.temmerman@mumc.nl, P. Debyelaan 25, 6229 HX Maastricht, the Netherlands, +31(0)433874633)

[1]Experimental Vascular Pathology Group, Department of Pathology, Cardiovascular Research Institute Maastricht, Maastricht University Medical Center, Maastricht, the Netherlands.

[2]Division of BioTherapeutics, Leiden Amsterdam Centre for Drug Research, Leiden University, Leiden, the Netherlands.

[3]Laboratory for Experimental Cardiology, University Medical Center Utrecht, Utrecht, the Netherlands.

[4]Institue of Microbiology and Immunology, National Yang-Ming University, Taipei 112, Taiwan

[5]Einthoven Laboratory for Vascular and Regenerative Medicine, Leiden University Medical Center, Leiden, The Netherlands.

[6]Department of Internal Medicine, Division of Thrombosis and Hemostasis, Leiden University Medical Center, Leiden, the Netherlands

[7]Department of Molecular Genetics, Cardiovascular Research Institute Maastricht, Maastricht University, Maastricht, the Netherlands.

[8]Department of Biochemistry, Cardiovascular Research Institute Maastricht, Maastricht University, Maastricht, the Netherlands.

[9]Centre for Cardiovascular Science, University of Edinburgh, Edinburgh, UK.

Gene	Forward primer (5'-3')	Reverse primer (5'-3')
Mcl-1	AAGAGGCTGGGATGGGTTTGT	AGTCCCCTATTGCACTCACAAG
HPRT	TTGCTCGAGATGTCATGAAGGA	AGCAGGTCAGCAAAGAACTTATAG
18S	GTAACCCGTTGAACCCCATT	CCATCCAATCGGTAGTAGCG

Supplementary Table 1: Primer sequences

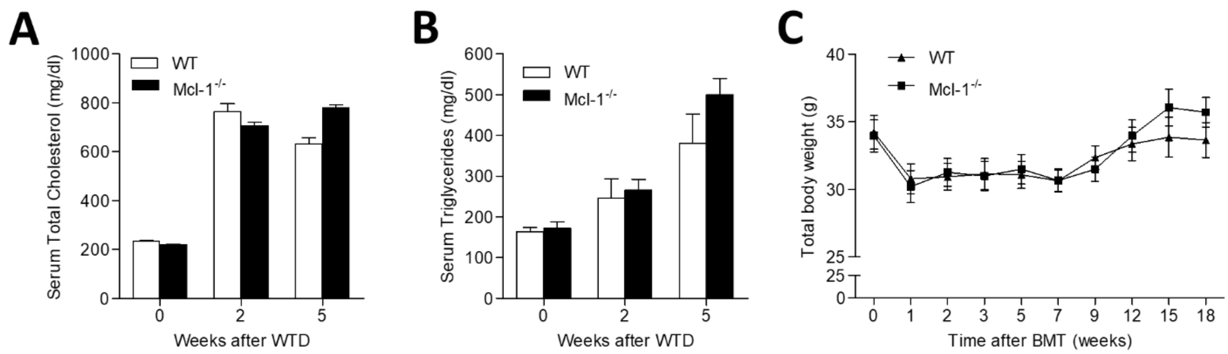

Supplementary Figure I. Lipid levels and total body weight of Mcl-1$^{-/-}$ chimeras are unchanged. (**A**) Serum total cholesterol levels at 0, 2 and 5 weeks after WTD. (**B**) Serum triglyceride levels at 0, 2 and 5 weeks after WTD. (**C**) Total body weight of WT and Mcl-1$^{-/-}$ chimeras. Data is presented as mean ± SEM. *:p<0.05

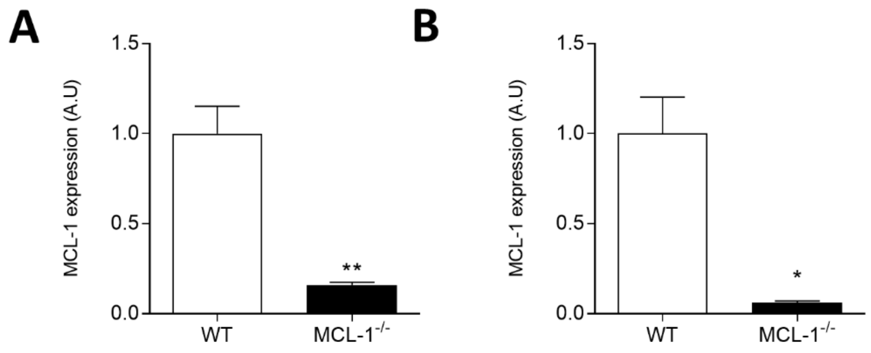

Supplementary Figure II: Mcl-1 gene knock-out confirmation by qPCR. Mcl-1 gene expression in BMDMs from Mcl-1$^{-/-}$ mice is almost blunted as compared to that of WT BMDM at day 7d (**A**) or 12d (**B**) of culturing. Data is presented as mean ± SEM. *:$p<0.05$; **:$p<0.01$.

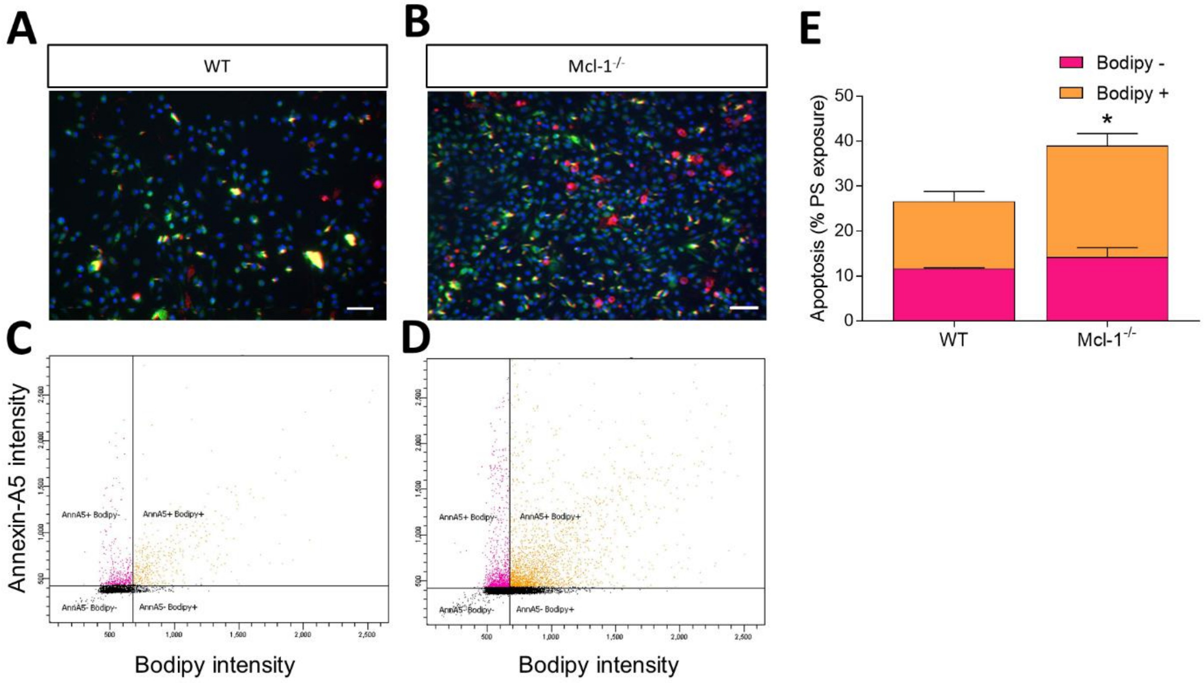

Supplementary Figure III: Lipid induced-apoptosis in Mcl-1$^{-/-}$ BMDMs. (**A**) WT and (**B**) Mcl-1$^{-/-}$ BMDMs were incubated for 2,5h with fluorescently-labeled oxLDL (bodipy, 20µg/ml) and subsequently stained for PS exposure using Alexa-647 labelled Annexin-A5 and cell associated fluorescence was measured by using a fluorescent microscope (BD pathway 855; representative image at 10X magnification). (**C**) and (**D**) show representative analysis performed with the DIVA software and (**E**) represents the quantification. Data is presented as mean ± SEM. *:$p<0.05$
